# Prevalence and Molecular Characterization of *Cryptosporidium* in Diarrheic Dairy Calves: A Descriptive Study in Family Farms of Southern Santa Catarina, Brazil

**DOI:** 10.3390/microorganisms14071552

**Published:** 2026-07-16

**Authors:** Guilherme Drescher, Larissa Américo, Bruna Costanski, Michail Sabino Moroz, Hanna Caroline Prochno, Fabiano Borges Figueiredo

**Affiliations:** 1 Carlos Chagas Institute, Oswaldo Cruz Foundation (Fiocruz), Curitiba 81310-020, PR, Brazil; contatobruna.vet@gmail.com; 2 Laboratory of Parasitology and Parasitic Diseases (LAPAR-CAV-UDESC), Department of Veterinary Medicine, Agroveterinary Sciences Center, Santa Catarina State University, Lages 88520-000, SC, Brazil; larissa_americo22@yahoo.com.br; 3 Graduate Program in Animal Science, School of Life Sciences, Pontifícia Universidade Católica do Paraná (PUCPR), Curitiba 80215-901, PR, Brazil; michailvet@outlook.com (M.S.M.); hannaprochno@yahoo.com.br (H.C.P.)

**Keywords:** *Cryptosporidium*, diarrhea, zoonosis, dairy cattle

## Abstract

This study describes the management practices and evaluates the molecular identification of *Cryptosporidium* species in dairy calves from southern Santa Catarina, Brazil, an important dairy cattle milk production region. Fecal diarrhea samples from 67 calves aged 1 to 90 days across 22 farms were analyzed by nested PCR for *Cryptosporidium* DNA detection. Sequencing 24 PCR-positive samples from 14 farms revealed the presence of *Cryptosporidium parvum*, demonstrating the circulation of the species in the farms. Phylogenetic analysis confirmed high sequence similarity (99.01–100%) with reference strains, indicating genetic diversity and homogeneity in infection dynamics. The identification of *C. parvum*, a zoonotic species, raises significant concerns for animal and public health. This research represents molecular evidence of the *Cryptosporidium parvum* in diarrheic dairy calves from farms in the southern part of Santa Catarina, Brazil, underscoring the need for targeted control strategies and ongoing molecular surveillance to mitigate transmission risks in dairy farming systems.

## 1. Introduction

Brazil has been ranked among the top six milk-producing countries, reaching around 24.5 million metric tons (MMT) in 2023 [1]. Santa Catarina State, especially the municipalities of Braço do Norte and São Ludgero, is a major milk-producing region and a recognized hub for Jersey cattle production [2]. The main obstacles of dairy farming are diseases that occur during the preweaning period in dairy calves. Diarrhea is one of the main causes of mortality in dairy calves [3]. One of the main causes of diarrhea is cryptosporidiosis, particularly affecting calves between 7 and 20 days of age [4,5].

Cryptosporidiosis is caused by the protozoan of the phylum Apicomplexa, and in cattle and other species including humans [6,7], the most isolated species is *C. parvum*, which causes bad absorptive diarrhea due to the destruction of enterocytes [8,9]. Molecular methods offer improved sensitivity and detection rates compared with oocyst detection by tinctorial microscopy and antigen detection by enzyme immunoassays (EIA) or immunochromatographic lateral flow assays (ICLF) [10,11]. Cattle are often infected by more than one *Cryptosporidium* species or genotype; therefore, caution needs to be exercised to consider poly-infection [10,12].

The presence of *Cryptosporidium* in calves’ feces has been studied in various countries [13], including several studies conducted in South America [14,15] and different Brazilian regions [16,17]. Important knowledge gaps persist regarding its prevalence, molecular characterization, and associated risk factors in important dairy-producing areas of Southern Brazil. In the southern region of Santa Catarina, a major hub for dairy production, particularly small- to medium-sized family farms, the occurrence and impact of cryptosporidiosis in dairy calves remain largely unknown. *Cryptosporidium* transmission is predominantly fecal-oral transmission on farms [18], and the strong association between infection rates and management practices, including proximity to water sources and inadequate waste disposal [19,20]. Moreover, the parasite’s high environmental resistance and the ineffectiveness of most disinfectants and chemotherapeutic agents make prevention particularly challenging [21,22].

Despite the significant importance of the Brazilian dairy sector, the prevalence of infection by *Cryptosporidium* spp. in dairy calves raised in this region remains unknown. The aim of this study was to evaluate the prevalence of cryptosporidiosis in dairy calves unresponsive to antibiotic therapy from the microregion of southern Santa Catarina (Brazil) and to map potential factors associated with the cryptosporidiosis prevalence.

## 2. Materials and Methods

### 2.1. Study Design and Data

Dairy farms were visited by a veterinarian once during the winter and spring of 2019, and were selected using a convenient sampling approach, focusing on properties reporting cases of diarrhea unresponsive to antibiotic therapy. Individual fecal samples were collected from 67 dairy calves, all of which were presented with diarrhea (Score 2 or 3/3 based in the McGuirk, 2008 [23]), at the time of sampling. Samples were collected through a partnership with a local agricultural cooperative in the study region. A single visit was conducted at each farm participating in the study to collect both samples and data. An optional questionnaire (open-ended, closed-ended, dichotomous, and multiple-choice questions) was collected through direct observation and interviews with the person responsible for calf rearing from each farm (Supplementary Material S1). Considering the limited sample size, management data were used only to characterize the farms included in the study, and no risk-factor analysis was carried out. The host factors (age and sex) and the environmental data location were the subjects of the data collection. In addition, management factors, such as management systems, herd size, presence of feed and water troughs, and source of water were collected.

### 2.2. Collection of Samples

A total of 22 small- to medium-sized family-dairy farms with no documented history of *Cryptosporidium* infection are in the southern of the Santa Catarina state, Brazil, in the municipalities of Orleans (n = 8), São Ludgero (n = 2), and Braço do Norte (n = 12), respectively Urchin Tracking Module (UTM) (28°21′32″ S, 49°17′27″ W), (28°19′33″ S, 49°10′36″ W) and (28°16′30″ S, 49°09′57″ W). Fecal samples were collected from Holstein (10%) and Jersey (90%) dairy calves, between 1 and 90 days of age, during the period from August to October 2019. At least 5 g of fecal material was directly obtained from the rectum of each calf using a single pair of latex gloves and placed in screw-topped specimen containers. The collected fecal samples were immediately placed in refrigerated conditions (4 °C) and transported to the Parasitology Laboratory of the University Barriga Verde (UNIBAVE), where they were stored at 4 °C until microscopic examination was performed.

### 2.3. Cryptosporidium Fecal Oocysts Identification

Microscopic examination for the detection of *Cryptosporidium* oocysts was performed on fecal suspensions using the modified Sheather’s sugar flotation technique [24], with fecal analysis conducted within a maximum of 24 h after sample collection. Modified Ziehl–Nielsen acid-fast staining was performed on the obtained concentrates. Oocyst identification was carried out at magnifications of ×40 to ×100. Positive samples for fecal oocysts identification were stored in 2.5% potassium dichromate at −20 °C until required for molecular analysis.

### 2.4. DNA Extraction and Purification

Fecal samples were submitted to the induction excystation process described by Ward and Wang (2001) [25]. Briefly, stored samples were transferred to 1.5 mL microcentrifuge tubes and resuspended in 500 µL of ultra-pure water. Posteriorly, samples were suspended in 100 µL of 1.5% taurocholic acid (Sigma-Aldrich, St. Louis, MO, USA) in water, vortexed for 5 min at Room Temperature (RT), and incubated at 37 °C for 2 h, vortexed for 30 s each 15 min, then centrifuged at 15,000× *g* for 5 min, RT. The resulting pellet was suspended in 500 µL lysis buffer [100 mM NaCl, 10 mM Tris-Cl, pH 8.0, 25 mM EDTA, 0.5% SDS, and 0.1 mg/mL proteinase K (Ludwig Biotec., Alvorada, RS, Brazil)], incubated at 56 °C for 2 h, vortexed for 30 seg each 15 min and the digest supernatant saved for DNA extraction. The analyses were carried out in the Laboratory of Cell Biology of the Carlos Chagas Institute (ICC/PR) in Curitiba—Brazil.

The phenol–chloroform method [26] was used for extracting PCR-ready DNA from fecal materials. Briefly, 150 µL of selected digested supernatants were extracted twice with phenol–chloroform–isoamyl alcohol (15:14:1) and the DNA precipitated with 1/10 volumes sodium acetate pH 8 ice-cold and 2 volumes of ice-cold ethanol. The DNA pellet was then dissolved in 20 µL ultra-pure water.

### 2.5. PCR Amplification and Subtyping

A two-step nested PCR protocol was used to amplify the 18S rDNA gene [27]. For the primary PCR, a PCR product of 763 bp was amplified using the forward primer 18SiCF2 (5′-GAC ATA TCA TTC AAG TTT CTG ACC-3′) (base pair position 292) and the reverse primer 18SiCR2 (5′-CTG AAG GAG TAA GGA ACA ACC-3′) (base pair position 1007). The PCR mixture consisted of a 3x PCR mixer containing buffer, MgCl_2_, Taq polymerase (Institute of Molecular Biology of Paraná—IBMP/FIOCRUZ, Curitiba, PR, Brazil), and 15 pmol of forward and reverse primers in a total 25 µL reaction mixture. Forty-five PCR cycles (94 °C for 30 s, 56 °C for 30 s, 72 °C for 30 s) were carried out in a thermocycler (Proflex, Thermo Fisher Scientific, Waltham, MA, USA) with an initial hot start (94 °C for 5 min) and a final extension (72 °C for 10 min). For the secondary PCR, a fragment of 587 bp was amplified using 1 µL of primary PCR product and nested forward 18SiCF1 (5′-CCT ATC AGC TTT AGA CGG TAG G-3′) (base pair position 289) and nested reverse 18SiCR1 (5′-TCT AAG AAT TTC ACC TCT GAC TG-3′) (base pair position 851) primers. The conditions for the secondary PCR were identical to those for the primary PCR, except the temperature of annealing, which was 58 °C. All reactions were performed using the negative control (reagent water) and the positive control (DNA of a previously sequenced *Cryptosporidium* isolate maintained at −20 °C in our laboratory); each sample was examined at least twice. The secondary PCR results from typical specimens were sequenced to validate the identification of *Cryptosporidium* species.

### 2.6. Detection of Amplified Products

The PCR amplification products were identified by running 10 µL of the PCR reaction mixture in 1.0% agarose gels in Tris-acetic acid-EDTA buffer, pH 8.0. Size markers were included on each gel. Gels were run at 90 V for 40 min, stained with ethidium bromide solution (Sigma-Aldrich, St. Louis, MO, USA) for 20 min, and PCR product bands were visualized under Ultraviolet (UV) light.

### 2.7. Sequencing Identification

The positive samples were identified using an ABI 3500xl DNA sequencing instrument (Applied Biosystems, Thermo Fisher Scientific, Waltham, MA, USA). Amplicons of positive DNA samples were detected by forward and reverse sequencing to guarantee the correctness of the sequences. A molecular phylogenetic tree was constructed using MEGA 7.0 software (Molecular Evolutionary Genetics Analysis, Pennsylvania State University, University Park, PA, USA) [28] with the General Time-Reversible (GTR) model and the maximum likelihood method. Before tree inference, the collected sequences were aligned with reference sequences using ClustalX 2.1 software (Conway Institute, University College Dublin, Dublin, Ireland) [29].

### 2.8. Statistical Analysis

Management practices were analyzed descriptively. Means, absolute frequencies, and relative frequencies were calculated for the main herd management variables, including the production system (semi-extensive, extensive, or intensive), type of farm operation (family-based farm, or pre-commercial farm), water source origin, availability of individual calf waterers, use of manure as fertilizer, and calf-rearing protocols.

## 3. Results

The average number of cows was 103 ± 49 (mean ± SD). Individual water troughs were observed in 78.6% of the farms (11/14), whereas 21.4% (3/14) used shared troughs. Regarding the management system of adult cattle on farms with *Cryptosporidium*-positive calves, 59.0% (n = 13) of the positive cases occurred under semi-extensive systems, whereas 41.0% (n = 9) were associated with intensive systems.

In the surveyed farms, water sources varied considerably, with spring water being the most common (42.9%, n = 6), followed closely by wells (35.7%, n = 5), while surface sources such as streams (7.1%, n = 1) or rivers/ponds (14.2%, n = 2) were also reported (Table 1).

### Prevalence of Cryptosporidium sp.

*Cryptosporidium* spp. oocysts were identified in the feces of 47.76% (32/67) of the examined calves using the modified Ziehl–Neelsen staining technique (Figure 1). We found that 68.18% (15/22) tested positive for *Cryptosporidium*. Among the 32 *Cryptosporidium*-positive calves, the highest prevalence was observed in 1-month-old animals (43.75%; n = 14), followed by 2-month-old (25.00%; n = 8) and 3-month-old calves (18.75%; n = 6). Lower positivity rates were detected in younger animals, with 9.38% (n = 3) at 15 days of age and only 3.13% (n = 1) at 10 days of age.

All 32 positive samples were subjected to DNA extraction. *Cryptosporidium* DNA was successfully detected in twenty-nine samples (90.6%), and the extracted DNA was quantified using the Qubit™ fluorometric method (Thermo Fisher Scientific, Waltham, MA, USA). However, DNA extraction was unsuccessful for 3 (9.38%) of the 32 samples due to low-quantity DNA and their sample was not sequenced. Subsequent PCR analysis confirmed *Cryptosporidium* sp. DNA in 24 (75%) of the 32 samples analyzed. From the 32 PCR-positive samples, 24 were selected for further molecular characterization to ensure geographic representation across the study region, including samples from different farms (Table 1).

These samples were subjected to sequencing to identify species and subtypes of *Cryptosporidium*. However, sequencing was unsuccessful for 9 (37.5%) of the 24 samples due to poor-quality reads or insufficient amplification, rendering their results inconclusive.

Sequencing analysis of the 15 (62.5%) successful samples (out of 24 initially selected) revealed the presence of *Cryptosporidium* species, which were used for downstream phylogenetic and subtype analyses (Table 2). These 15 samples originated from 14 different properties where sequencing was performed Genbank submission no. (SUB16279819). *Cryptosporidium parvum* was confirmed in samples from all the farms, highlighting its prevalence in these settings. These findings demonstrate the homogeneity of *Cryptosporidium* species distribution within and across farms, underscoring the complexity of infection dynamics in cattle populations.

Genomic DNA of oocysts were isolated from dairy calves containing oocysts and amplified with 18S rRNA directed nested-PCR, and obtained sequences were used for BLASTn analyses available at the National Center for Biotechnology Information (NCBI) (https://blast.ncbi.nlm.nih.gov/Blast.cgi, accessed on 10 January 2026). The samples showed similarity of 99.01 to 100% to the *C. parvum* species. The samples showed similarity to the *Cryptosporidium* species, with 13 (86.7%) with *C. parvum* (GenBank accession number PQ338760.1) and 2 (13.3%) with *C. parvum* (KX198143.1).

In nine samples, the products generated in the sequencing could not be analyzed using bioinformatics tools, and it was not possible to confirm whether they were *Cryptosporidium* species.

## 4. Discussion

This study showed that the prevalence of *Cryptosporidium parvum* in dairy calves with diarrhea was 48%. These results are aligning with reports from other Brazilian studies, such as those documenting rates between 40% and 60% in calves [16,17], and echo global patterns observed in dairy herds across South America, Europe, and North America, where prevalences often range from 30% to 70% depending on age cohorts and diagnostic methods [13,14,15]. The present study showed that the prevalence of *Cryptosporidium* was not different of the others study in dairy cattle in Brazil and other countries around the world [17,30,31,32,33,34,35].

Notably, the confirmation of *Cryptosporidium* DNA in 75% of Microscopy-positive samples via PCR highlights potential discrepancies in diagnostic sensitivity, yet reinforces the pathogen’s pervasive presence, particularly in pre-weaned calves vulnerable to diarrheal syndromes. These findings were obtained from small- to medium-sized family-based dairy farms in the Southern region of the Santa Catarina state, Brazil, which frequently lack adequate infrastructure and biosecurity measures (such as dedicated calving areas, improved hygiene facilities, or proper manure management) [36]. Such limitations likely exacerbate transmission risks among animals through increased environmental contamination and fecal-oral exposure in confined or semi-confined settings. Such elevated infection rates likely stem from intensive rearing practices, fecal-oral transmission routes, and suboptimal sanitation measures, as evidenced by prior associations with farm management variables [37], and emphasize the urgent need for enhanced biosecurity protocols to curb economic losses in milk production while mitigating zoonotic risks to human health [6,38].

Based on our findings, we observed that most farms rely on untreated water sources, primarily originating from natural supplies such as springs and wells. These untreated or minimally protected sources are prone to contamination with minerals (e.g., excess iron, manganese, sulfates, or sodium), organic matter, or pathogens, which can compromise the quality of reconstituted milk replacer, potentially reduce calf intake, impair rumen development, and increase susceptibility to digestive disorders. Environmental contamination, including unchanged bedding on the farm, contributed to infection persistence, consistent with findings on the resilience of *Cryptosporidium* and reinfection potential due to its ubiquitous nature [39,40]. In resource-limited smallholder systems, where infrastructure for water treatment or testing is often absent, this oversight may undermine the intended advantages of transitioning toward milk replacers.

The PCR analysis in this study provided critical molecular insights into *Cryptosporidium* infections among dairy calves, revealing a positivity rate of 75% (24 out of 32 selected microscopy-positive samples) and enabling species identification through subsequent sequencing of all representative amplicons, of which 15 (62.5%) yielded interpretable data. Notably, the distribution of *Cryptosporidium* species appeared to correlate with calves age, with *C. parvum* predominantly detected in younger and older animals, aligning with its known propensity for causing acute infections in pre-weaned calves whose immature immune systems render them particularly susceptible [41,42].

Of the samples successfully sequenced, *Cryptosporidium parvum* was identified. *C. parvum* is the most prevalent species in young calves in the pre-weaning phase (<2 months old) and post-weaned [33,43]. The dairy calves included in this research were of different ages, and symptomatic; however, the symptoms of cryptosporidiosis in cattle are dependent on the infecting species and the host’s immune status [32]. The sequencing outcome was likely influenced by technical challenges inherent to molecular analysis of fecal specimens, including the presence of PCR inhibitors, the use of phenol–chloroform extraction instead of commercial kits with inhibitor-removal columns, and prolonged storage at −20 °C prior to molecular processing [10,44].

The prevalence observed in the present study (48%) is comparable to figures reported in international studies. In Brazil, the intensive dairy farming systems prevalent in several regions have been associated with the circulation of zoonotic *C. parvum* subtypes. This finding underscores the potential for animal-to-human transmission through environmental contamination and occupational exposure [14,34]. This scenario aligns with previous Brazilian studies that identified contamination of water sources on dairy farms with zoonotic *C. parvum* subtypes, highlighting the role of cattle as reservoirs for environmental contamination and the potential for waterborne transmission to humans [20,31]. In this context, the presence of diarrheic calves shedding high numbers of oocysts represents a significant source of environmental contamination, as these structures can be dispersed through runoff, soil, and untreated water sources commonly used in the studied region [18,38]. Nevertheless, the precise pathways by which oocysts gain access to water sources remain insufficiently characterized in this region. Whether contamination occurs through direct fecal deposition, inadequate manure management, or rainfall-induced runoff has yet to be elucidated. Consequently, further investigations are essential to identify these transmission routes and enable the implementation of effective interruption strategies [20,31].

The sequencing results from 15 successfully amplified fecal samples derived from 14 (63.6%) dairy farms demonstrated the circulation of *C. parvum*. Specifically, BLASTn alignments of the 18S rRNA gene amplicons revealed all samples exhibiting 99.01–100% sequence identity to *C. parvum* reference strains. *C. parvum* predominates in pre-weaned calves, potentially reflecting age-dependent differences in host susceptibility, oocyst environmental persistence, and dam-to-calf transmission dynamics [38,42]. In a 2023 Western European follow-up study across 51 dairy farms in Belgium, France, and the Netherlands, co-detection of *C. parvum* (23–25% prevalence), emphasizing vertical and horizontal transmission dynamics exacerbated by intensive rearing practices, with no significant association to housing type or seasonality but clear zoonotic implications from *C. parvum* [38,41,42].

This study provides important insights into the characterization of *Cryptosporidium* species present in diarrheic dairy calves within a key milk-producing region in the Santa Catarina state of Brazil. Nevertheless, certain limitations should be addressed to enhance scientific rigor. This study has some limitations that should be considered when interpreting the results. The limited number of farms and animals included, together with the sampling strategy used, may have affected the precision and representativeness of the prevalence estimates. Therefore, the prevalence reported here should be interpreted with caution. In addition, fecal samples were collected only once from each animal, which may have reduced the ability to detect true-positive calves, particularly if oocyst shedding was intermittent or below the detection limit at the time of sampling. Finally, although management information was collected, this was a descriptive observational study and did not have sufficient sample size or statistical power to robustly evaluate associations between management practices and *Cryptosporidium* infection. For this reason, management data were used only to characterize the sampled farms, and no risk-factor analysis was performed. Future studies with larger sample sizes, repeated sampling, and designs specifically planned to evaluate risk factors are needed. Sequencing was performed on a strategically selected subset rather than all PCR-positive samples, limiting the resolution of population genetic diversity and co-infection dynamics. Epidemiological inferences and intervention recommendations require validation through larger-scale management-integrated studies that combine comprehensive sampling with farm-level husbandry assessments.

## 5. Conclusions

The findings of this study highlight the circulation of *Cryptosporidium parvum* in farms located in the southern part of the Santa Catarina in Brazil. The presence of *C. parvum*, a zoonotic species that is primarily cattle-adapted, raises important implications for both animal health and public health.

## Figures and Tables

**Figure 1:**
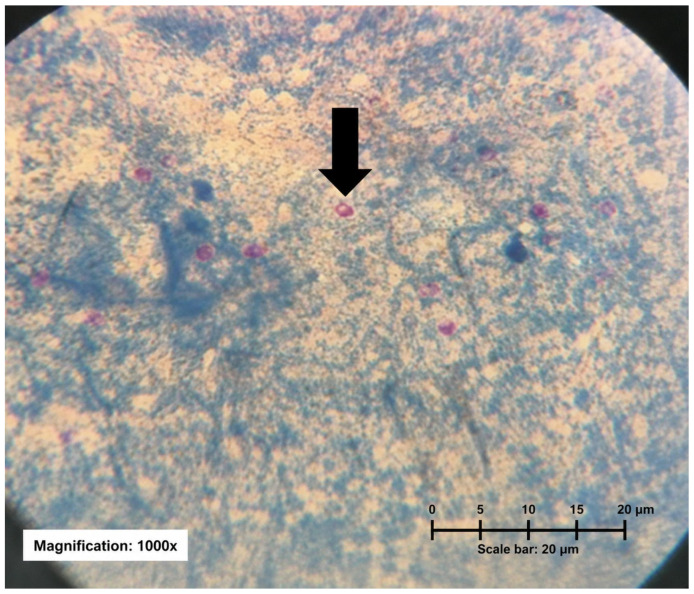
Detection *Cryptosporidium* spp. oocysts in fecal samples from diarrheic calves. Modified Ziehl–Neelsen-stained fecal smear showing *Cryptosporidium* spp. oocysts (arrow) in diarrheic calves under oil immersion (1000×).

**Table 1:** Descriptive summary of management and housing characteristics of 22 small- to medium-sized farms visited during winter and spring 2019 in southern Santa Catarina, Brazil.

Information	% (n)
System ^1^	
Semi-extensive	47.6% (10)
Intensive	23.8% (5)
Extensive	28.6% (6)
Type of farm operation ^2^	
Family-based farm	94.4% (17)
Pre-commercial farm	5.6% (1)
Water source ^3^	
Stream	7.1% (1)
Spring	42.9% (6)
Well	35.7% (5)
River and/or pond	14.2% (2)
Use of animal manure as fertilizer ^4^	
Yes	100.0% (21)
No	0.0% (0)
Type of milk used to feed calves ^4^	
Whole milk	52.4% (11)
Waste milk	23.8% (5)
Milk replacer	4.8% (1)
Whole milk + Milk replacer	19.0% (4)
Milk allowance ^5^ (L/day)	
5	26.3% (5)
4	63.2% (12)
3	10.5% (2)
Housing system ^2^	
Individual	78.6% (11)
Group	21.4% (3)

^1^ n = 21; ^2^ n = 18; ^3^ n = 14; ^4^ n = 21; ^5^ n = 19. Because the questionnaire was optional, some farms were unaware of it and/or did not complete all questions, resulting in missing data for specific items.

**Table 2:** Molecular identification of *Cryptosporidium* species in microscopy-positive fecal samples collected from diarrheic dairy calves on small- to medium-sized predominantly family-based farms during winter and spring 2019 in southern Santa Catarina, Brazil.

Sample ID	Farm	City	Age (d)	Microscopy	PCR	Sequencing
1	A	Braço do Norte	90	+	*Cryptosporidium* sp.	*Cryptosporidium parvum*
6	B	Braço do Norte	30	+	*Cryptosporidium* sp.	*Cryptosporidium parvum*
7			30	+	*Cryptosporidium* sp.	**
8			60	+	*Cryptosporidium* sp.	*Cryptosporidium parvum*
14	F	Braço do Norte	5	+	*Cryptosporidium* sp.	*Cryptosporidium parvum*
15	G	Braço do Norte	10	+	*Cryptosporidium* sp.	**
16			15	+	*Cryptosporidium* sp.	*Cryptosporidium parvum*
20			30	+	*Cryptosporidium* sp.	*Cryptosporidium parvum*
26	I	Braço do Norte	60	+	*Cryptosporidium* sp.	**
30			30	+	*Cryptosporidium* sp.	**
32	J	Braço do Norte	30	+	*Cryptosporidium* sp.	**
33	K	Braço do Norte	90	+	*Cryptosporidium* sp.	*Cryptosporidium parvum* *
36			60	+	*Cryptosporidium* sp.	*Cryptosporidium parvum*
38	L	Braço do Norte	30	+	*Cryptosporidium* sp.	**
41	N	São Ludgero	15	+	*Cryptosporidium* sp.	*Cryptosporidium parvum*
42			60	+	*Cryptosporidium* sp.	*Cryptosporidium parvum*
44			30	+	*Cryptosporidium* sp.	*Cryptosporidium parvum*
47	O	Orleans	30	+	*Cryptosporidium* sp.	*Cryptosporidium parvum*
51	P	Orleans	30	+	*Cryptosporidium* sp.	**
54			30	+	*Cryptosporidium* sp.	*Cryptosporidium parvum*
60	S	Orleans	30	+	*Cryptosporidium* sp.	Cryptosporidium parvum *
61	T	Orleans	60	+	*Cryptosporidium* sp.	*Cryptosporidium parvum*
63	V	Orleans	90	+	*Cryptosporidium* sp.	*Cryptosporidium parvum*
66			90	+	*Cryptosporidium* sp.	*Cryptosporidium parvum*

* *C. parvum* (Sequence: KX198143.1). ** Not correctly sequenced. d: days.

## Data Availability

The original contributions presented in this study are included in the article/Supplementary Materials. Further inquiries can be directed to the corresponding authors.

## Supplementary Materials

The following supporting information can be downloaded at: https://www.mdpi.com/article/10.3390/microorganisms14071552/s1, Questionnaire S1: Standardized Epidemiological Questionnaire. Reference [45] is cited in the Supplementary Materials.
