# Supplementary material for: Prevalence and Molecular Characterization of Cryptosporidium in Diarrheic Dairy Calves: A Descriptive Study in Family Farms of Southern Santa Catarina, Brazil
Source: Microorganisms. 2026 Jul 16;14(7):1552. doi: 10.3390/microorganisms14071552 (PMC13413461; doi:10.3390/microorganisms14071552)
Supplement: Supplementary file 1 [file microorganisms-14-01552-s001.zip › microorganisms-4364548-supplementary.pdf]

Supplementary Materials

Standardized Epidemiological Questionnaire

This epidemiological questionnaire was applied during on-site visits to farms included in the study.

Farm Identification

Farm identification number: ________________________________

Farm name: ____

Owner: ____

Telephone: ____

Geographical location (GPS coordinates): ____________________

Farm size (hectares): _______________________________________

Total number of cattle: _____________________________________

Production purpose: ( ) Dairy ( ) Beef

Number of calves younger than 6 months of age: _____________

Number of animals presenting diarrhea: _____________________

Management system: ( ) Extensive ( ) Semi-intensive ( ) Intensive

Production system: ( ) Family-based ( ) Pre-commercial ( ) Commercial

Source of drinking water for cattle:

( ) River ( ) Spring ( ) Pond ( ) Treated piped water ( ) Other: _____________

On-farm drinking water reservoir: ( ) Yes ( ) No

Use of manure as fertilizer: ( ) Yes ( ) No

Hygienic–sanitary conditions: ( ) Excellent ( ) Good ( ) Fair ( ) Poor

Type of milk feeding: ( ) Discarded milk ( ) Whole milk ( ) Milk replacer

Daily milk volume offered (L/day): _________________________

Deworming practices: ( ) Yes ( ) No

If yes, describe protocol (product, frequency, dose): ______

Antimicrobial treatment used in diarrhea cases: ____________

Calf management practices: ( ) Adequate ( ) Moderate ( ) Inadequate

Source: Adapted from Almeida (2006).

ALMEIDA, A. J. Diagnóstico E Fatores De Risco Da Criptosporidiose Bovina Na Microrregião De Campos Dos Goytacazes-Rj, E Identificação De Cryptosporidium Parvum Pela Reação Em Cadeia Da Polimerase (Pcr). 67 f. Tese (Doutorado) - Universidade Estadual do Norte Fluminense Darcy Ribeiro – Uenf, Campos dos Goytacazes – Rj, 2006.

Individual Animal Data

Animal 1

Animal identification number: ____________________________

Sex: ( ) Female ( ) Male

Age (days): ____

Fecal consistency: ( ) Normal ( ) Soft ( ) Watery

Fecal color: ____

Duration of diarrhea (days): _____________________________

Animal 2

Animal identification number: ____________________________

Sex: ( ) Female ( ) Male

Age (days): ____

Fecal consistency: ( ) Normal ( ) Soft ( ) Watery

Fecal color: ____

Duration of diarrhea (days): ______________________________

Animal 3

Animal identification number: _____________________________

Sex: ( ) Female ( ) Male

Age (days): ____

Fecal consistency: ( ) Normal ( ) Soft ( ) Watery

Fecal color: ____

Duration of diarrhea (days): ______________________________

Animal 4

Animal identification number: _____________________________

Sex: ( ) Female ( ) Male

Age (days): ____

Fecal consistency: ( ) Normal ( ) Soft ( ) Watery

Fecal color: ____

Duration of diarrhea (days): ______________________________

…
